# Supplementary material for: Complexome profiling of the Chlamydomonas psb28 mutant reveals TEF5 as an early PSII assembly factor
Source: Plant Cell. 2025 Mar 17;37(6):koaf055. doi: 10.1093/plcell/koaf055 (PMC12164590; doi:10.1093/plcell/koaf055)
Supplement: koaf055_Supplementary_Data [file koaf055_supplementary_data.zip › psb28_interactive.zip!/plots/intensity/qualityPlots/peptides/_Cre02.g078507.t1.2.html]

### Cre02.g078507.t1.2

Trivial name:   
SeqCoverage [median]: 24.70  
Mass: 24.37 kDa  
  
 

| Sequence | MissedCleavage | Mass [Da] | wtIntensity | muIntensity | Score |
| --- | --- | --- | --- | --- | --- |
| NQYTTVSGVPVAVLDSEYAR | 0 | 2168.07 | 968.00 | 0.00 | 132.62 |
| SVQQADANYNAMSK | 0 | 1525.68 | 2294.69 | 3854.31 | 148.62 |
| VVLVNIDQAK | 0 | 1097.64 | 0.00 | 617.52 | 67.99 |
|  |  |  | 3262.7 | 4471.8 |  |
